# Fractionation of Glycomacropeptide from Whey Using Positively Charged Ultrafiltration Membranes

**DOI:** 10.3390/foods7100166

**Published:** 2018-10-09

**Authors:** Abhiram Arunkumar, Mark R. Etzel

**Affiliations:** Department of Chemical and Biological Engineering, University of Wisconsin, 1605 Linden Dr., Madison, WI 53706, USA; arunkumar@uwalumni.com

**Keywords:** sieving coefficient, phenylketonuria, polyhexamethylene biguanide

## Abstract

Fractionation of the bovine glycomacropeptide (GMP) from the other proteins in cheese whey was examined using ultrafiltration membranes surface modified to contain positively charged polymer brushes made of polyhexamethylene biguanide. By placing a strong positive charge on a 1000 kDa ultrafiltration membrane and adjusting the pH of whey close to the isoelectric point of GMP, a 14-fold increase in selectivity was observed compared to unmodified membranes. A one stage membrane system gave 90% pure GMP and a three-stage rectification system gave 97% pure GMP. The charged membrane was salt-tolerant up to 40 mS cm^−1^ conductivity, allowing fractionation of GMP directly from cheese whey without first lowering the whey conductivity by water dilution. Thus, similarly sized proteins that differed somewhat in isoelectric points and were 50–100 fold smaller than the membrane molecular weight cut-off (MWCO), were cleanly fractionated using charged ultrafiltration membranes without water addition. This is the first study to report on the use of salt-tolerant charged ultrafiltration membranes to produce chromatographically pure protein fractions from whey, making ultrafiltration an attractive alternative to chromatography for dairy protein fractionation.

## 1. Introduction

Whey proteins are used for a variety of purposes; in snack foods, infant formula, food for the elderly, and for fitness enthusiasts. Specific whey proteins such as glycomacropeptide (GMP) are also used in foods for people with medical conditions like phenylketonuria (PKU). Indeed, GMP has been examined in great detail for the nutritional management of PKU, given that it is the only protein in nature that does not contain the amino acid phenylalanine, and hence, can be used as a protein source for patients suffering from PKU [1,2].

Chromatography is the common method for fractionation of GMP from whey. Several studies examined the use of chromatography to fractionate GMP from whey [2,3,4,5,6]. In addition to chromatography, other methods examined are precipitation or enzymatic degradation of the non-GMP whey proteins followed by ultrafiltration [7,8,9]. Past work used uncharged ultrafiltration membranes rated 20–50 kDa to obtain GMP in the permeate [7]. However, these tight membranes had low permeation rates of GMP, requiring diafiltration to recover more GMP. The process of diafiltration washes not only GMP into the permeate, but also the other whey proteins such as alpha-lactalbumin (ALA) and beta-lactoglobulin (BLG), which reduces the purity of the GMP.

Charged ultrafiltration membranes were examined for protein fractionation in the biopharmaceutical industry because of the simplicity of operation and benefits of true linear scalability and economy of scale compared to chromatography [10,11]. Other benefits result because ultrafiltration does not require use of salts and buffers as does process chromatography. Past work in our laboratory used a stirred cell containing a 30 kDa positively charged ultrafiltration membrane to fractionate GMP from the other whey proteins (OWP) [12]. This required dilution of the whey to lower its conductivity to 4 mS cm^−1^ (two parts water to one part whey) to increase the selectivity. Moreover, the 30 kDa ultrafiltration membrane had a low sieving coefficients (*S_o_*) of GMP (*S_o_* = 0.32) and OWP (*S_o_* = 0.024). Low sieving coefficients of GMP require diafiltration to increase the recovery of GMP in the permeate.

Water dilution is one approach to lower the conductivity of the feed solution and increase selectivity by enhancing electrostatic interactions during protein ultrafiltration [12,13,14,15]. Another approach is to increase the surface charge density on the membrane pores to allow increased conductivity of whey. The hypothesis of the present work was that a high charge density imparted by using a polycation polymer brush to modify the membrane surface would allow using whey without water dilution to adjust conductivity. This makes it possible to electrostatically exclude the other whey proteins such as ALA and BLG, which are positively charged at a pH < 4, and permeate GMP which is nearly uncharged at pH < 4. The occurrence of maximum protein transmission when pH = pI is well explained by Burns and Zydney [16] and has been observed by several research groups [17,18,19,20,21,22].

Different membrane staging options (one-stage and three-stage) were examined for fractionation of GMP from whey using these salt tolerant ultrafiltration membranes. This is the first study to report on the use of a salt-tolerant tangential flow ultrafiltration membrane for fractionation of proteins from a real dairy fluid such as cheese whey at its natural conductivity (8–12 mS cm^−1^), without dilution and without diafiltration. Elimination of the added water used for dilution and diafiltration and its resulting wastewater generation might make ultrafiltration membranes an environmentally friendly alternative to chromatography for the fractionation of proteins other than those found in cheese whey. Furthermore, data were obtained using a membrane module that is a linear scale model of membrane modules currently in place in industry, offering a route for direct industrial scale-up of the results of this study.

## 2. Experimental

### 2.1. Membrane Modification Using Polyhexamethylene Biguanide (PHMB)

Pellicon-2^®^ Mini membrane modules (EMD Millipore, Billerica, MA, USA) made of composite regenerated cellulose (Ultracel™ PLC) were used having a molecular weight cut-off (MWCO) of 10 kDa (PLCGC), 100 kDa (PLCHK), 300 kDa (PLCMK) or 1000 kDa (PLCXK), and each incorporating suspended screens (Viscous screens or V-screens) as feed solution spacers. The modules had a length of 21 cm and a retentate hold-up volume (*V_HR_*) of 17 cm^3^.

Membranes charge-modified using polyhexamethylene biguanide (PHMB) were made following the procedure of Riordan et al. [23] and Arunkumar and Etzel [24]. The reaction consisted of four steps: (1) recirculation of 0.3 M NaOH in 30% dimethyl sulfoxide (DMSO) for 2 h to create hydroxyl groups, (2) recirculation of 7.5% (*v*/*v*) allyl glycidyl ether (AGE) in 0.3 M NaOH in 30% (*v*/*v*) DMSO for 48 h to convert the hydroxyl groups into allyl groups, (3) recirculation of 20 g L^−1^ *N*-bromosuccinimide (NBS) in 30% DMSO for 2 h to brominate the allyl groups, and (4) recirculation of 15% *w*/*w* solution of PHMB at pH 12 for 24 h to substitute the amine for bromine and present the biguanide group on the surface of the membrane (see supplement for chemical structures). The membranes were rinsed using 100 L m^−2^ of deionized (DI) water and the AGE solution changed every 24 h in step (2). The membranes were rinsed using DI water after completion of steps (2), (3), and (4). The membrane hydraulic permeability was measured before and after charge modification and was found to decrease by about 50% for the 1000 kDa membrane.

Membranes charge-modified using quaternary amine (Q) were made using the procedure of van Reis [25] and Arunkumar and Etzel [18]. Briefly, the membrane was recirculated with 10 mol m^−2^ of 3-bromopropyl trimethyl ammonium bromide for 21 h, and then rinsed with 1% acetic acid for 2 h prior to use.

### 2.2. Measurement of Protein Sieving Coefficients

Swiss cheese whey from the Babcock Hall Dairy Plant, University of Wisconsin was stored with 0.05% NaN_3_. Prior to ultrafiltration, whey was centrifuged at 4000× *g* and 0 °C for 1 h and the butter-fat separated whey vacuum filtered through Whatman 4 filter paper (GE Healthcare, Pittsburgh, PA, USA). Adding 1 M HCl adjusted the pH to 3.5 where the conductivity was 10–12 mS cm^−1^.

Protein sieving coefficients of GMP and OWP were measured under total recycle conditions. Prior to taking a sample for analysis, whey was recirculated through the membrane for 12–15 h at a tangential flow rate of 1200–1320 mL min^−1^ equal to a flux of 720–790 L m^−2^ h^−1^ (LMH) and permeate returned to the feed solution container.

After ultrafiltration, membranes were rinsed with 100 L m^−2^ of DI water at 22 °C and then cleaned using: (1) DI water at 50 °C, (2) 1 g L^−1^ pepsin in 50 mM phosphoric acid, 2 M NaCl at pH 2.5 for 4 h, (3) DI water at 50 °C, (4) 0.2% Tween-20^®^ overnight, (5) acid wash solution of 1% *v*/*v* Ultrasil^®^-75 (Ecolab, St. Paul, MN, USA) at pH 2.5 for 4 h, and (6) a final flush using DI water at 40 °C. In most cases, the membrane hydraulic permeability was restored after cleaning, but in few of the experiments, the hydraulic permeability declined by no more than 20%. 

### 2.3. Staging Experiments

**Figure 1:**
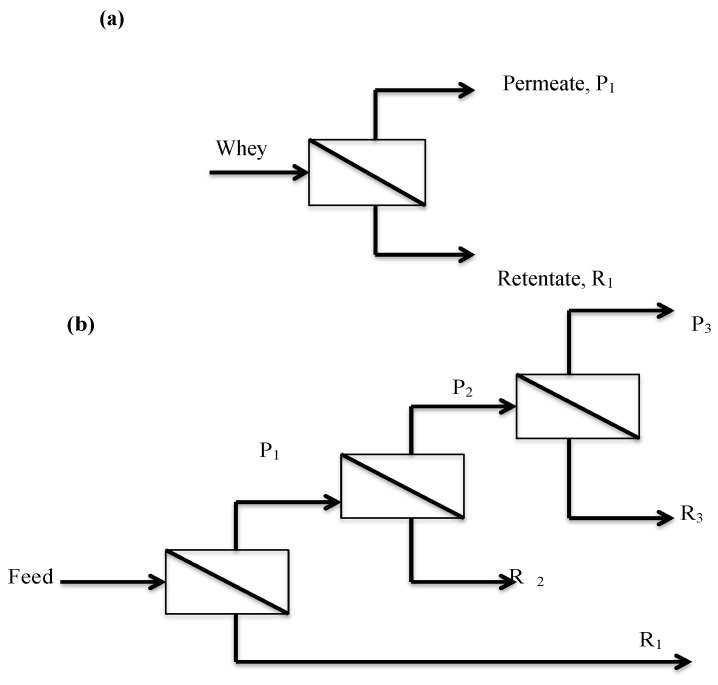
Flow configurations evaluated: (**a**) one-stage concentration, and (**b**) three-stage rectification. The membranes used in Stages 1 to 3 were the same.

Staging experiments were performed using the 1000 kDa PHMB membrane only. Two staging configurations were used. The first system (Figure 1a) was a simple one-stage ultrafiltration system with 90% of the feed stream collected as permeate, P_1_. The second system was a three-stage rectification system [17] (Figure 1b). Feed solution was pumped into the membrane and 90% of it was collected as permeate (P_1_). Permeate, P_1_ was then pumped again into the same charged ultrafiltration membrane, and 90% of it was collected as permeate, P_2_. Permeate P_2_ was pumped into the same charged ultrafiltration membrane and then 90% of it was collected as permeate (P_3_). Retentate samples from each stage were pooled together and analyzed for GMP and OWP. 

Prior to submission of samples for amino acid analysis, permeate samples were concentrated 10-fold using a 10 kDa ultrafiltration membrane (Ultracel™ PLC, PLCGC, Pellicon-2™ Mini), diafiltered using 50 mM H_3_PO_4_ at pH 3.5, and lyophilized. GMP and peptide impurities are strongly retained by the membrane at this pH.

### 2.4. Analysis of Protein Concentrations

Analyses of permeate and retentate samples were performed by HPLC. Samples were analyzed for GMP and OWP separately. Whey contained 1.1 ± 0.2 g L^−1^ GMP and 5.4 ± 0.7 g L^−1^ OWP. For GMP, the procedure of Doultani et al. [3] was used. Briefly, the OWP were precipitated using 7.2% trichloroacetic acid and 2.5% Na_2_SO_4_, centrifuged at 4000× *g* for 1 h, and the desalted supernatant injected into a BioSil SEC-125^®^ (Bio-Rad, Hercules, CA, USA) size-exclusion column. The mobile phase was 50 mM sodium phosphate, pH 3 and the flow rate was 0.5 mL min^−1^. The area at 214 nm was used quantify GMP using a calibration standard.

For OWP, cation exchange chromatography was used. The OWP are positively charged at pH 4 and bind to a cation-exchanger at pH 4 while GMP passes through unbound. A 1 mL HiTrap SPFF column (GE Healthcare) was connected to an AKTA^®^ Explorer 100 chromatography system (GE Healthcare). The chromatographic procedure consisted of 4 steps: (1) equilibration of the column using 10 mM sodium lactate, pH 4 (buffer A), (2) injection of 2 mL of sample, (3) washing out unbound material and (4) eluting the bound OWP using buffer B (buffer A +1 M NaCl). The peak area at 280 nm was used to calculate the OWP concentrations using a calibration standard. Sodium dodecyl sulfate polyacrylamide gel electrophoresis (SDS-PAGE) and laser densitometry were also used to quantify ALA and BLG separately in some cases.

### 2.5. Phenylalanine Analysis

Samples from the different staging systems were analyzed for phenylalanine (Phe). Analysis was conducted at the Experiment Station Chemical Laboratories (ESCL), University of Missouri, Columbia, MO, USA. Association of Official Analytical Chemists (AOAC) method 982.30 was used for obtaining an amino acid profile (AAP) [26].

## 3. Results

The present work evaluated ultrafiltration membranes modified with the salt-tolerant ligand PHMB to purify GMP from the OWP in cheese whey without water dilution. Water dilution of whey is needed using ultrafiltration membranes modified with the traditional quaternary amine (Q) ligand [12,17,18]. One goal of the present work was to avoid the addition of water in order to avoid its concomitant burden on the environment.

### 3.1. Sieving Coefficients Measured Using Different Charged and Uncharged Membranes

Sieving coefficients of GMP and OWP at total recycle were measured using different ultrafiltration membranes (Table 1). The goal was to have the smaller GMP permeate the membrane while retaining the larger OWP. This goal is met when the ratio of proteins in the permeate and retentate are different, making the ratio of the sieving coefficients (also known as the selectivity *ψ*) also different. The uncharged 10 kDa ultrafiltration membrane failed to meet this goal because the sieving coefficients of OWP and GMP were about the same and both were less than *S_o_* = 0.05. The 10 kDa membrane was too tight. Conversely, the 100 kDa uncharged membrane was too loose, having high sieving coefficients of OWP (*S_o_* = 0.34) and GMP (*S_o_* = 0.75), and the same low selectivity as the 10 kDa membrane (*ψ* = 2.2, *p* > 0.05). The 1000 kDa uncharged membrane was the worst, having the highest sieving coefficients of OWP (*S_o_* = 0.67) and GMP (*S_o_* = 1.03), and the lowest selectivity (*ψ* = 1.54). In conclusion, all three uncharged membranes failed to fractionate GMP from the OWP. 

The 300 kDa membrane containing the Q ligand was a considerable improvement compared to all the uncharged membranes. At a permeate flux of *J_v_* = 12 LMH, the 300 kDa Q membrane mostly permeated GMP (*S_o_* = 0.919) and mostly retained the OWP (*S_o_* = 0.31), and the selectivity (*ψ* = 3.1) was moderately (41%) higher than for the 100 kDa uncharged membrane (*p* = 0.05). Increasing the permeate flux to *J_v_* = 33 LMH, increased the selectivity to *ψ* = 5.7 or 160 % higher than the 100 kDa uncharged membrane. Thus, placing a positive charge on the ultrafiltration membrane met the goal of fractionating GMP from the OWP.

The 1000 kDa PHMB membrane was the most successful. The smaller GMP permeated the membrane (*S_o_* = 0.61) while the larger OWP was retained (*S_o_* = 0.029). The selectivity *ψ* = 21 was nearly 10-fold higher than for the 100 kDa uncharged membrane.

### 3.2. Effect of Salt Using the 1000 kDa PHMB Membrane

The effect of adding NaCl to the whey on the separation performance of the 1000 kDa PHMB membrane is shown in Table 2. The hypothesis was that adding salt to the feed solution will lead to shielding of charges between the membrane and proteins, thereby increasing the sieving coefficients of GMP and the OWP and reducing selectivity. The sieving coefficients of ALA, GMP, and the OWP were measured at pH 3.5 using different salt concentrations. OWP includes ALA, BLG and the other whey proteins larger than GMP. From 50 to 1000 mM, the sieving coefficient of GMP decreased, while that of the OWP increased (*p* < 0.05). This was attributed to a slight increase in the sieving coefficient of ALA. Increasing the salt concentration from 50 to 1000 mM caused an 83% decrease in the selectivity from *ψ* = 21 to *ψ* = 3.6. Nevertheless, the selectivity of the 1000 kDa PHMB membrane at 1000 mM NaCl was more than double that of the 1000 kDa unmodified membrane (*ψ* = 1.54), an indication of strong salt-tolerance of the PHMB ligand.

### 3.3. Effect of Staging Configuration

Two different staging configurations were evaluated using the 1000 kDa PHMB membrane: (1) one-stage concentration, and (2) three-stage rectification (Figure 1). The flow of mass in each configuration was calculated from the measured concentrations and volumes of each stream, and put on a basis of 100 g of total protein entering in the feed stream (Table 3 and Table 4).

It is useful to understand the flow of mass through the membranes stages using the sieving coefficients: *S_o_* GMP = 0.61 and *S_o_* OWP = 0.029, and the volume concentration factor (*VCF*) for each stage. When *VCF* = 10 then 90% of the feed solution passes through the membrane into the permeate. For example, for the one stage system, 2000 mL of Feed Solution (FS) formed 1800 mL of P_1_ and 200 mL of *R*_1_. For the three stage system, 1800 mL of P_1_ formed 1620 mL of P_2_ and 180 mL of *R*_2_, and 1620 mL of P_2_ formed 1458 mL of P_3_ and 162 mL of *R*_3_. The three retentate streams were mixed together (*R*_mix_ = *R*_1_ + *R*_2_ + *R*_3_) and analyzed at the end of the experiment. 

For example, in the one stage system (Table 3), permeate P_1_ contained only 1% OWP, but 77% GMP. Conversely, retentate *R*_1_ contained 95% of the OWP and only 37% of the GMP. In one stage, the mass ratio of GMP to OWP increased 157-fold from 0.15 in the FS to 9.1 in stream P_1_. Permeate (P_1_) had 90% pure GMP on a total protein basis (GMP + OWP) as measured by HPLC and 2.0 mg Phe g^−1^ dry protein as measured by AAP.

In the three stage system (Table 4), this process is repeated using permeate from the previous stage as the feed stream to the next higher stage. By analogy to distillation, the more volatile light component (*S_o_* > 0.5) flows “upwards” to the top stage permeate, and the less volatile heavy component (*S_o_* < 0.5) flows “downwards” to the retentate. The light component was GMP and the heavy component was OWP. This was observed as permeate P_3_ contained only 0.2% of the heavy component OWP, but 24% of the light component GMP. Conversely, the retentate mixture *R*_mix_ contained 95% of the OWP. The GMP purity increased monotonically stage-by-stage from 18.8% in the *FS* to 97% in stream P_3_ that had 1.4 mg Phe g^−1^ dry protein by AAP analysis.

The overall mass balance was calculated by comparing the mass of GMP or OWP fed to the system to the mass flowing out in the permeate and retentate steams. Mass balance closure averaged 105% for the one stage system (Table 3), and 95% for the three stage system (Table 4). Thus, the proteins in the feed stream were accounted for either in the permeate or the retentate. This is an important point because there were no protein losses in the membrane cascade, and no water was used in the separation.

## 4. Discussion and Conclusions

This work examined the use of salt tolerant positively charged ultrafiltration membranes to fractionate GMP from undiluted cheese whey. Previous work on fractionation of GMP from whey used chromatography [2,3,4,5,6], enzymatic methods [9], precipitation [8] or tight ultrafiltration membranes (30 kDa or 50 kDa) [7]. Positively charged membranes of 30 kDa were examined, but required dilution of the whey to adjust the conductivity to 4 mS cm^−1^ to enhance electrostatic repulsion for the separation in addition to the membrane being too tight for GMP (*S_o_* = 0.30) [12]. Chromatography requires dilution of the whey to adjust its conductivity so that the dynamic binding capacity of the charged stationary phase for GMP is high. Furthermore, chromatography uses added water and buffer salts to wash out unbound material and elute bound protein. Water added during chromatography ends up as wastewater and increased thermal emissions to the environment because the final protein is sold as a dry powder. The purpose of the current work was to fractionate GMP from whey without dilution of the whey and without added water. This was accomplished by developing salt-tolerant charged ultrafiltration membranes.

Riordan et al. [23] examined the use of salt tolerant membrane adsorbers for protein purification. They found that ligands incorporating the biguanide functionality on chromatography supports significantly increased protein binding capacity compared to the quarternary ammonium functionality. The ligand, PHMB, is a polycation polymer brush that contains 12 biguanide groups per chain. Therefore, functionalizing an ultrafiltration membrane with PHMB should strongly increase the surface charge density of the membrane pores and make the ultrafiltration membrane less sensitive to increases in the feed solution conductivity. This is important because elevated conductivities do occur in biological feed solutions (2–40 mS cm^−1^) and a charged ultrafiltration membrane sensitive to these conductivities would not be useful for protein fractionation.

### 4.1. Sieving Coefficients as a Function of Membrane MWCO and Charge

As shown in Table 1, uncharged ultrafiltration membranes were not useful for fractionation of GMP from whey. It might be expected that a 10 kDa ultrafiltration membrane would have a high selectivity for GMP, given that GMP is only 8.6 kDa in molecular mass while the other whey proteins are larger (14.4–150 kDa). This was not observed; rather, the 10 kDa ultrafiltration membrane was too tight, mostly rejecting both GMP and OWP. The 100 kDa unmodified membrane offered no better selectivity as the 10 kDa uncharged membrane (*p* > 0.05). A 300 kDa positively charged membrane was required in order to have a high sieving coefficient of GMP (*S_o_* = 0.78) and a low sieving coefficient for OWP (*S_o_* = 0.13), giving a selectivity of 5.7 at a flux of *J_v_* = 33 LMH. Based on the mass balance models derived in our previous work [18], this selectivity was still inadequate to give more than 95% pure GMP in a three stage rectification system. The 1000 kDa PHMB membrane had a high transmission of GMP (*S_o_* = 0.61) and almost completely rejected the OWP (*S_o_* = 0.029), giving a selectivity *ψ* = 21.

### 4.2. Variation of Sieving Coefficients with Ionic Strength for the 1000 kDa PHMB Membrane

NaCl was added to whey at pH 3.5 to examine the salt-tolerance of the 1000 kDa PHMB membrane. Sieving coefficients of ALA, GMP and OWP were measured (Table 2). OWP includes ALA, BLG and the other larger whey proteins that absorb at 280 nm. It is observed that between 50 to 1000 mM, the sieving coefficient of GMP decreased, while that of the OWP increased (*p* < 0.05). This was attributed to a slight increase in the sieving coefficient of ALA, and GMP aggregation as salt content increased. Because GMP was close to its isoelectric point, increased ionic strength might cause aggregation by isoelectric precipitation. This hypothesis was supported using dynamic light scattering, where the GMP hydrodynamic diameter was found to increase as the ionic strength increased.

### 4.3. Ultrafiltration Cascades for GMP Fractionation from Whey

Two flow configurations were evaluated using the 1000 kDa PHMB membrane and a *VCF* = 10: (a) one-stage concentration, and (b) three-stage rectification. Permeates were highly pure as measured by HPLC: 90% for the one-stage system, corresponding to 2 mg Phe g^−1^ of dry protein, and 97% for the three-stage system, corresponding to 1.4 mg Phe g^−1^ of dry protein. This is significant because commercially available GMP at 2.0 mg Phe g^−1^ protein and 94% GMP is lower in purity [5], and is manufactured using an ultrafiltration and diafiltration process, with the permeate from the ultrafiltration process polished using ion-exchange chromatography. The present work is first to show that PKU-grade purity of GMP can be obtained using a charged 1000 kDa PHMB ultrafiltration membrane without chromatography, dilution of the whey, diafiltration, or added water. This is also the first study to use salt-tolerant ultrafiltration membranes for protein fractionation. Salt-tolerant ultrafiltration membranes might be an environmentally friendly alternative to chromatography for the fractionation of proteins other than those found in cheese whey.

## Figures and Tables

**Table 1:** Sieving coefficient (*S_o_*) of glycomacropeptide (GMP) and other whey proteins (OWP), and selectivity (*ψ*) measured using different ultrafiltration membranes. Experiments performed at pH 3.5 and 22 °C and the natural conductivity of whey unless mentioned otherwise. Values are average ±95% CI for duplicate experiments unless mentioned otherwise.

Membrane	LMH	*S_o_* GMP	*S_o_* OWP	ψ
10 kDa unmodified	12	0.039 ± 0.02	0.016 ± 0.007	2.3 ± 0.1
100 kDa unmodified (*n* = 5, pH 6.8)	24	0.75 ± 0.06	0.34 ± 0.06	2.2 ± 0.3
1000 kDa unmodified	27	1.03 ± 0.03	0.67 ± 0.02	1.54 ± 0.08
300 kDa Q (pH = 3)	12	0.919 ± 0.007	0.31 ± 0.09	3.1 ± 0.9
	33	0.71 ± 0.03	0.126 ± 0.008	5.7 ± 0.2
1000 kDa PHMB	30	0.61 ± 0.10	0.029 ± 0.000	21 ± 3

LMH: permeate flux in L m^−2^ h^−1^; *S_o_*: Sieving coefficient; GMP: glycomacropeptide; OWP: other whey proteins; Q: quaternary amine; PHMB: polyhexamethylene biguanide; ψ: selectivity = *S_o_* GMP/*S_o_* OWP.

**Table 2:** Effect of added salt on the sieving coefficients (*S_o_*) of GMP, alpha-lactalbumin (ALA), and the OWP, and the GMP-OWP selectivity (*ψ*) using whey at pH 3.5 and 22 °C, and a permeate flux *J_v_* = 30 L m^−2^ h^−1^ (LMH). Values are average ±95% CI for duplicate experiments.

Added NaCl (mM)	Conductivity (mS cm^−1^)	*S_o_* GMP	*S_o_* ALA	*S_o_* OWP	ψ
whey = 0	10	0.6 ± 0.1	__	0.029 ± 0.000	21 ± 2
50	16	0.8 ± 0.1	0.022 ± 0.001	0.036 ± 0.003	21 ± 1
500	67	0.4 ± 0.2	0.16 ± 0.008	0.05 ± 0.01	8 ± 5
1000	124	0.26 ± 0.04	0.18 ± 0.01	0.072 ± 0.007	3.6 ± 0.5

ALA: alpha-lactalbumin.

**Table 3:** Mass balances for GMP and the OWP, and purity of GMP for the one-stage flow configuration evaluated in this work.

Stream	GMP (g)	OWP (g)	Purity (%)
Feed Solution (FS)	12.9 ± 0.1	87.1 ± 0.1	12.9 ± 0.1
Permeate (P_1_)	10.0 ± 0.5	1.1 ± 0.2	90 ± 2
Retentate (*R*_1_)	5 ± 1	83 ± 6	5 ± 2
(P_1_ + *R*_1_)/FS	114 ± 6%	96 ± 6%	

**Table 4:** Mass balances for GMP and the OWP, and purity of GMP for the three-stage flow configuration evaluated in this work.

Stream	GMP (g)	OWP (g)	Purity (%)
Feed Solution (FS)	18.9 ± 0.4	81.1 ± 0.4	18.9 ± 0.4
Permeate (P_1_)	7.6 ± 0.6	3 ± 2	72 ± 9
Retentate (*R*_1_)	9 ± 2	65 ± 17	11.9 ± 0.6
Permeate (P_2_)	5 ± 1	1.9 ± 0.3	73 ± 8
Retentate (*R*_2_)	4 ± 1	6 ± 7	43 ± 39
Permeate (P_3_)	4.4 ± 0.7	0.1 ± 0.2	97 ± 3
Retentate (*R*_3_)	1.5 ± 0.3	2 ± 2	51 ± 33
Retentate mix (*R*_mix_)	13 ± 3	77 ± 1	15 ± 3
(P_3_ + *R*_mix_)/FS	94 ± 9%	95.0 ± 0.9%	

## Supplementary Materials

The following are available online at http://www.mdpi.com/2304-8158/7/10/166/s1, Figure S1: Structures for immobilization chemistry.
