# Supplementary figures and images for: Fractionation of Glycomacropeptide from Whey Using Positively Charged Ultrafiltration Membranes
Source: Foods. 2018 Oct 9;7(10):166. doi: 10.3390/foods7100166 (PMC6210718; doi:10.3390/foods7100166)

### Supplementary Materials:

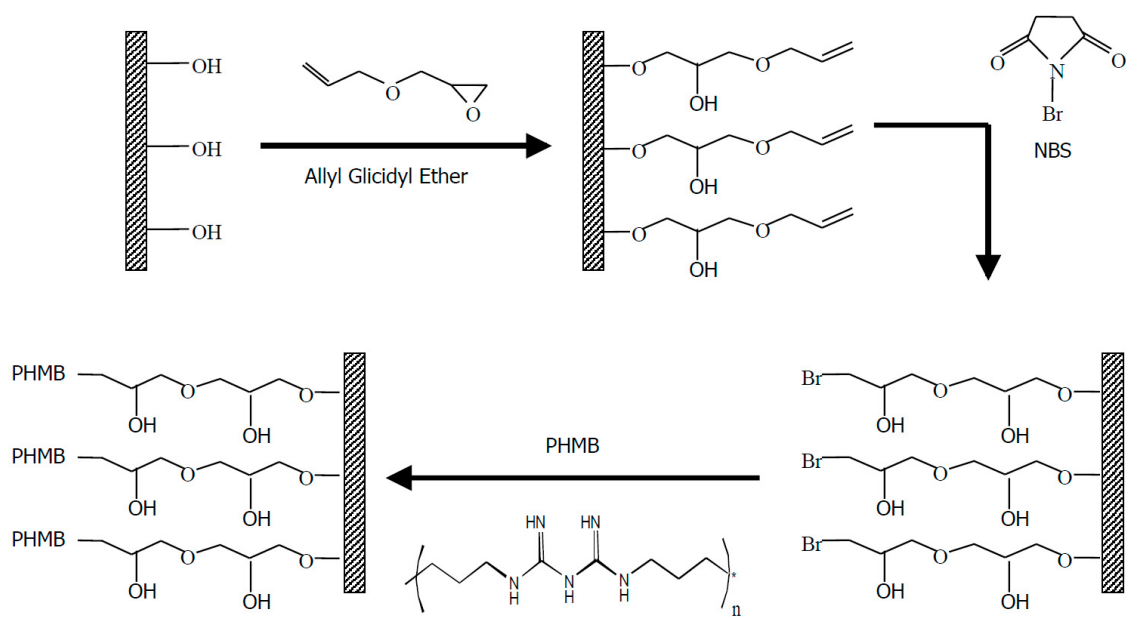

**Figure S1.** Structures for immobilization chemistry.

Supplement: Supplementary file 1 [file foods-07-00166-s001.pdf]
